# Evaluating health related quality of life in outpatients receiving anti-cancer treatment: results from an observational, cross-sectional study

**DOI:** 10.1186/s12955-021-01876-9

**Published:** 2021-10-18

**Authors:** Hae-Jin Suh Oh, Ángeles Flórez Menéndez, Víctor Sacristán Santos, Ángeles Rodríguez Martínez, Francisca Fernández Ribeiro, Lucía Vilanova-Trillo, Manuel Constenla Figueiras, Manuel Pereiro Ferreiros

**Affiliations:** 1 Dermatology Department, Pontevedra University Hospital, Simón Bolívar s/n, 36003 Pontevedra, Spain; 2 Medical Oncology Department, A Coruña University Hospital, Coruña, Spain; 3 Medical Oncology Department, Pontevedra University Hospital, Pontevedra, Spain; 4 Pharmacy Department, Pontevedra University Hospital, Pontevedra, Spain; 5 Surgical Medical Specialties Department, Dermatology Section, Santiago de Compostela University, Santiago, Spain

**Keywords:** Cancer, Cutaneous adverse events, Targeted therapies, Patient-reported outcomes, FACT-G, Quality of life

## Abstract

**Background:**

The aim of the study was to assess health-related quality of life (HRQOL) in outpatients receiving anti-cancer treatment.

**Methods:**

Observational, cross-sectional, single-center study that assessed HRQOL in cancer patients receiving antineoplastic treatment.

**Results:**

A total of 184 patients were included in the study; the median total FACT-G score was 66 ± 12.9; the scores for the physical well-being, social/family well-being, emotional well-being and functional well-being domains were 17.8 + 4.8, 19.1 ± 4.4, 14.8 ± 3.8 and 14.3 ± 4.7 respectively. Patients with adverse events had poorer HRQOL compared to those without them (FACT-G score 62.2 vs. 67.3; *p* < 0.05). In the multivariate analysis the variables associated with poorer HRQOL in the form of a gradient were tumor stage and performance status (ECOG); female sex was also associated with poorer HRQOL.

**Conclusion:**

In our study, the neoplastic disease and anti-cancer treatment toxicities had an impact on HRQOL. Patients had poorer scores in the functional well-being domain and higher ones in the social/family well-being domain. Variables associated with worse HRQOL were tumor stage, performance status (ECOG) and female sex.

## Background

In general, health-related quality of life (HRQOL) encompasses the subjective perceptions of the positive and negative aspects of disease, including physical, emotional, social and cognitive functions and, in particular, disease symptoms and treatment side effects. In last decades, HRQOL has increasingly become a major focus in cancer studies and the information on this particular topic is increasingly required by medical agencies in the context of approval of new treatment options [1–3]

A patient-reported outcome (PRO) is a measurement of any aspect of a patient's health status that is retrieved directly from the patient. The information captured by a PRO instrument can provide first-hand evidence of the benefit or detriment of health status from the patient’s perspective and helps to identify specific issues that may modify treatment decisions and provide guidance for determining an appropriate and personalized care [4–6].

In clinical research, the Functional Assessment of Cancer Therapy-General (FACT-G) has been validated and one of the most commonly used PRO instruments to assess HRQOL in cancer patients. It can be used to objectively quantify issues in domains that are not routinely screened along the treatment in cancer patients. Early recognition of HRQOL issues in specific domains of FACT-G that are not consistently examined in routine care helps to improve patient satisfaction, increase survival rates, reduce hospitalizations, and decrease costs to the healthcare system [7–9].

Although PRO instruments are usually incorporated in clinical trials, there is not extensive data regarding overall HRQOL in daily clinical practice. Therefore, the main objective of the current study was to assess HRQOL in outpatients receiving anti-cancer treatment in daily clinical practice using the Functional Assessment of Cancer Therapy-General (FACT-G) Questionnaire. Secondary objectives were to evaluate the impact of adverse events (AEs) and to explore possible predictors of quality of life.

## Methods

### Study design

Cross-sectional, observational study conducted in a single centre between April and December 2018.

### Study population and recruitment

A total consecutive sampling of patients meeting eligibility criteria (age equal to or greater than 18 years old, any solid tumor under active antineoplastic treatment, which include conventional chemotherapy, targeted therapy and immunotherapy) was performed at the Medical Oncology Service of the University Hospital Center (CHU) Pontevedra, Spain. Patients receiving radiotherapy at the time of initial evaluation and those not able to answer PRO questionnaires were excluded.

Physicians and nurses at the Medical Oncology Service Day Hospital and at the hospital dispensing office of cancer drugs carried out recruitment of patients.

### Study procedures and variables

Informed consent was obtained from study participants before performing any study procedure. A medical oncologist evaluated patients that met the eligibility criteria. Patients that developed cutaneous adverse events (CAEs) were additionally evaluated by a dermatologist. Detailed history and examination were performed to confirm CAEs and classify them according to usual clinical practice.

To assess patient overall HRQOL, the FACT-G questionnaire was used. The FACT-G comprises an overall score (scale range 0–108, higher score reflects better quality of life) and 4 subscale scores: physical well-being (PWB, range 0–28), social/family well-being (SFWB, range 0–28), emotional well-being (EWB, range 0–24), and functional well-being (FWB, range 0–28). The necessary license for the use of the FACT-G questionnaire was obtained. A prior Spanish version of FACT-G showed good reliability and validity to be used as a HRQOL tool among Spanish-speaking patients [10, 11]. Patients completed paper FACT-G questionnaires.

The ECOG (Eastern Cooperative Oncology Group) Scale of Performance Status (PS) was used to quantify the functional status of patients [12]. Tumor stage was determined using the American Joint Committee on Cancer (AJCC) TNM classification [13].

Data on demographic and clinical characteristics were collected. This was done through an interview with the participants, as well as with the review of their medical history. CTCAE (version 4.03) was used to determine the severity of AEs [14].

Targeted therapies were considered all those that act against specific molecular targets, monoclonal antibodies and immunotherapies. All classic antineoplastic drugs were considered non-targeted therapies.

### Statistical analysis

Stata V12.0 statistical software (Stata Corporation, College Station, TX, USA) was used for statistical analysis.

### Descriptive analysis

The clinical and sociodemographic characteristics of the sample were described using measures of central tendency and dispersion in the case of quantitative variables, as well as frequency tables and distribution of percentages in the case of qualitative variables.

Patients with different levels of HRQOL were compared using statistical hypothesis testing (Student t-test, Mann–Whitney U test). For all tests the level of significance was set to *p* = 0.05 (level adjusted according to Bonferroni procedure when necessary). Chi square was performed in case of categorical data with Fisher´s exact test when needed.

General quality of life, measured by the FACT-G questionnaire, was examined as well as its association with severity and number of adverse events, general condition of the patient, type of tumor, tumor stage, type of treatment and number of cycles received by the patient. To achieve this, contrast tests of media differences were used for continuous variables of normal distribution (ANOVA) and non-normal distribution (Kruskal–Wallis).

Possible HRQOL predictors were studied using bivariate and multivariate linear regression models. For the inclusion of the variables in the models, the results of the bivariate analysis (variables with *p* value < 0.250), the theoretical sense and the sample size were taken into account. The comparison between models was made using the Aikaike information criterion (AIC) and Bayesian information criterion (BIC) methods.

Effects of possible confounding factors (type of tumor, preventive treatment, type of antineoplastic treatment, general condition, age and sex) were controlled using multivariate analysis.

## Results

### Characteristics of the study population

A total of 201 patients were eligible for the study, and 17 declined to participate. Thus, 184 patients were included in the study: 103 (56%) women and 81 (44%) men; mean age was 60.5 + 11.8 and the most frequent tumors were gastrointestinal cancer (38%) followed by breast (25.5%) and lung (15.8%) cancer; 103 (56%) patients received targeted therapy and 81 (44%) conventional chemotherapy. The majority of patients presented with tumor stage IV (77.7%) and most of the patients included in our study were symptomatic but completely ambulatory (77.2%); 126 (68.4%) patients had previous medical conditions (hypertension, diabetes or dyslipemia (72.2%), depression and anxiety (13.4%), hypothyroidism (11.2%), others (3.2%)). Demographic and clinical characteristics of study population are summarized in Table 1.

**Table 1:** Demographic and clinical characteristics

Variable	Total n = 184
Gender, n (%)	
Male	81 (44.0)
Female	103 (56.0)
Age at diagnosis, years	
Mean (SD)	60.5 (11.8)
Median (IQR)	63.2 (51.4–70.3)
Tumor type, n (%)	
Gastrointestinal	70 (38.0)
Breast	47 (25.5)
Lung	29 (15.8)
Urological/renal	18 (9.8)
Gynecologic	9 (4.9)
Other	11 (6.0)
Tumor stage, n (%)	
Stage II	15 (8.1)
Stage III	26 (14.1)
Stage IV	143 (77.7)
Type of treatment, n (%)	
Conventional chemotherapy	81 (44.0)
Targeted therapy	103 (56.0)
Previous lines of treatment, n	
Mean (SD)	1.44 (0.96)
Median (IQR)	1 (1–2)
Treatment duration, months	
Mean (SD)	6.8 (6.8)
Median (IQR)	4 (2–8.5)
Previous medical conditions	
No	58 (31.5)
Yes	126 (68.4)
ECOG Performance Status	
0 (Asymptomatic)	9 (4.9)
1 (Symptomatic, but completely ambulatory)	142 (77.2)
2 (Symptomatic, < 50% of time in bed)	30 (16.3)
3 (Symptomatic, > 50% of time in bed)	3 (1.6)

SD, standard deviation; IQR, interquartile range

### Adverse events

In our study, 142 (77.2%) patients developed AEs and 42 (22.8%) did not have any AE; 114 patients (61.9%) developed cutaneous AEs (CAEs), 73 patients (39.6%) presented non cutaneous AEs (NCAEs); total number of AEs was 260 (177 CAEs and 83 NCAEs); 126 (68.5%) patients had one AE and 58 (31.5%) had two or more concomitant AEs; most patients had grade 1 or 2 AEs (69.2% and 25.8% respectively). Characteristics of AEs are summarized in Table 2.

**Table 2:** Adverse Events (AEs)

Variable	Total (%)
Patients that presented AEs, n (%)	
Yes	142 (77.2)
No	42 (22.8)
Total number of AEs	260
Type of AEs, n (%)	
Asthenia	35 (13.5)
Pruritus	29 (11.2)
Xerosis	24 (9.2)
Palmar-plantar erythrodysesthesia (PPE)	24 (9.2)
Alopecia	21 (8.0)
Neurotoxicity	20 (7.7)
Papulopustular rash	17 (6.5)
Gastrointestinal toxicity	16 (6.2)
Ungual apparatus alterations	13 (5.0)
Pigmentary changes	13 (5.0)
Others*	48 (18.5)
Number of AEs per patient, n(%)	
Patients with one AE	126 (68.5)
Patients with two or more AEs	58 (31.5)
Severity of AEs, n (%)	
Grade 1	180 (69.2)
Grade 2	67 (25.8)
Grade 3	13 (5.0)
Grade 4	–
Treatment interruption due to AEs	
Yes	20 (10.9)
No	164 (89.1)

^*^Others: photosensitivity, arthromyalgia, hematological toxicity, mucositis, dysgeusia, headache, folliculitis, trichomegaly, eyelid edema, purpura, other erythematous cutaneous reactions

### Quality of life indices

**Fig. 1:**
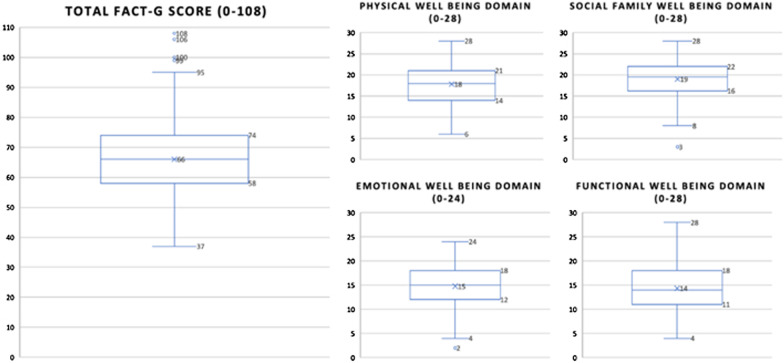
Total FACT-G score and Physical Well Being, Social Family Well Being, Emotional Well Being and Functional Well Being domain subscale scores in outpatients receiving anti-cancer treatment

All patients included in the study (N = 184) answered the FACT-G questionnaire, with an average score of 66 ± 12.9. The scores for the domains were as follows: 17.8 + 4.8 in physical well-being (PWB); 19.1 ± 4.4 social/family well-being (SFWB); 14.8 ± 3.8 in emotional well-being (EWB); 14.3 ± 4.7 in functional well-being (FWB) (Fig. 1).

Patients that presented AEs had significantly poorer HRQOL compared to patients that did not present any AE (total FACT-G score 62.2 vs. 67.3; *p* = 0.03). Regarding domain data, there were no differences in SFWB and FWB, but patients with AEs presented poorer HRQOL in PWB and EMB domains (Table 3).

**Table 3:** FACT-G scores according to the presence of AEs

FACT scores	Patients with AEs (N = 142)	Patients without AES (N = 42)	*p* value
FACT-G total (0–108) Mean (95% CI)	62.2 (60.0–64.3)	67.3 (63.7–70.8)	0.03
PWB (0–28) Mean (95%CI)	16.2 (15.4–17.1)	19.5 (18.1–20.7)	< 0.01
SFWB (0–28) Mean (95%CI)	19.1 (18.5–19.7)	18.3 (17.0–19.5)	0.21
EWB (0–24) Mean (95%CI)	13.1 (12.4–13.7)	15.9 (14.8–16.9)	< 0.01
FWB (0–28) Mean (95%CI)	13.7 (12.9–14.5)	13.7 (12.3–15.0)	0.94

### Quality of life predictors

General HRQOL, measured by the FACT-G questionnaire, was not associated to the type of tumor, type of treatment, duration of treatment, or the number of previous treatment lines received. On the contrary, an association was found between HRQOL and tumor stage (*p* = 0.02), the general condition measured by the ECOG PS (*p* = 0.0001), and the number and severity of AEs (*p* = 0.03 and *p* = 0.01, respectively).

Bivariate and multivariate analysis results showed that variables associated with HRQOL were tumor stage, general condition measured by the ECOG performance status and female sex. The following variables were not associated with HRQOL: appearance of CAEs, type of treatment, type of tumor, duration of treatment, number of previous lines of treatment, presence of concomitant disorders and age (Table 4.)

**Table 4:** Quality of life determinants in bivariate and multivariate analysis

Variables	Bivariate β [95% CI] (p value)	Multivariate β [IC 95%] (p value)
Cutaneous adverse events	−1.88 [−5.73; 1.98] (0.339)	
Type of treatment		
Conventional chemotherapy	1	
Targeted therapy	−0.98 [−4.76; 2.80] (0.610)	
Type of tumor		
Breast	1	
Digestive	1.63 [−3.20; 6.47] (0.505)	
Lung	−2.14 [−8.19; 3.91] (0.485)	
Gynecological	−2.21 [−11.5; 7.11] (0.640)	
Urological	0.18 [−6.92; 7.28] (0.961)	
Other	0.60 [−7.98; 9.18] (0.891)	
Tumor stage		
2	1	1
3	−11.4 [−19.4; −3.42] (0.005)	−10.18 [−17.38; 2.99] (0.006)
4	−12.0 [−18.7; −5.29] (0.001)	−11.59 [−17.66; −5.52] (< 0.0001)
Number of treatment cycles 1–5 6–10 11–15 16–20 More than 20	1 5.75 [0.92; 10.58] (0.020) 4.82 [−1.95; 11.60] (0.162) 1.11 [−7.66; 9.88] (0.804) 2.55 [−3.89; 9.00] (0.436)	
Performance status (ECOG) 0 1 2 3	1 −14.37 [−22.41; −6.32] (0.001) −22.47 [−31.36; −13.57] (< 0.0001) −36.0 [−51.60; −20.40] (< 0.0001)	1 −14.63 [−22.26; −7.01] (< 0.0001] −21.28 [−29.72; 12.85] (< 0.0001) −34.11 [−48.91; −19.31] (< 0.0001)
Performance status (ECOG)		
0	1	1
1	−14.37 [−22.41; −6.32] (0.001)	−14.63 [−22.26; −7.01] (< 0.0001]
2	−22.47 [−31.36; −13.57] (< 0.0001)	−21.28 [−29.72; 12.85] (< 0.0001)
3	−36.0 [−51.60; −20.40] (< 0.0001)	−34.11 [−48.91; −19.31] (< 0.0001)
Previous treatment lines		
1		
2	−0.76 [−6.54; 5.03] (0.796)	
More than 2	−6.96 [−15.07; 1.15] (0.092)	
Concomitant disorders	−2.35 [−6.38; 1.68] (0.252)	
Age	−0.05 [−0.21; 0.10] (0.502)	
Female sex	−4.62 [−8.34; −0.89] (0.015)	−4.01 [−7.40; −0.63] (0.020)

## Discussion

Quality of life assessment in cancer patients has become an important factor to consider in a scenario in which successful treatment is measured not only in terms of overall survival or progression free survival, but also in terms of HRQOL maintenance or improvement. Multiple PRO instruments are usually incorporated in clinical trials or clinical research, they are either cancer specific, treatment specific or symptom specific, but there is not extensive data regarding overall HRQOL in daily clinical practice.

The purpose of our study was to evaluate general quality of life in outpatients receiving anti-cancer therapy by means of FACT-G, a well-known PRO instrument in the context of cancer research. This questionnaire specifically developed for cancer patients has gone through many validation studies and is one of the most commonly used tools to assess HRQOL in cancer patients and survivors [7–9]. A previous Spanish version of FACT-G demonstrated good reliability and validity to be used as a HRQOL instrument among Spanish-speaking patients [10, 11].

In our study, the median total and subdomain FACT-G scores were similar to the results reported by Abu Sharour et al. (total FACT-G score 65.79 + 12.03; PWB 16.94 + 7.32; SFWB 18.6 + 4.59; EWB 14.83 + 5.35; FWB 12.36 + 7.03).They evaluated quality of life in cancer patients during their treatment phase, and as in our study, the highest scores were obtained in the SFWB domain and the lowest in the FWB and EWB domains[15].

Jacob et al. also evaluated HRQOL among cancer patients with advanced disease and reported a total FACT-G score of 62.4 + 10.0, with the lowest score in the FWB domain as well (9.3 + 3.8); in their study the most important predictor of lower quality of life was financial difficulty [16].

Regarding AEs, in our study patients that developed AEs had significantly poorer quality of life compared to patients without AEs (total FACT-G score 62.2 *v*s 67.3; p = 0.03), and differences were also statistically significant in PWB and EWB domains. Likewise, other studies that evaluated the impact of different AEs on quality of life found that PWB and EWB domains had the greatest negative impact [17–20].

Although we cannot make direct comparisons with other studies due to the different PRO instruments used to evaluate HRQOL, the use of antineoplastic agents is usually associated to adverse events and these are known to have an impact on patients' HRQOL [21–23].

In our study, the most frequent AEs were asthenia or cancer-related fatigue, pruritus,xerosis, palmar-plantar erythrodysestesia and alopecia. Cancer-related fatigue can be present in 48–75% of cancer patients and it is one of the most frequent symptoms reported by these patients and has a well known impact on HRQOL [23, 24].

Pruritus and xerosis can be major AEs associated with EGFR inhibitors and in a study carried out by Clabbers et al. pruritus and xerosis were reported by the patients as the most impactful AEs[25].

Palmar-plantar erythrodysestesia or hand foot syndrome may appear with various molecules and can affect up to 50% of patients receiving capecitabine [26].

A study performed by Urakawa et al. concluded that this AE had a stronger impact on HRQOL compared to other skin toxicities of chemotherapy [27].

Finally, alopecia can be related to conventional chemotherapy, targeted therapy or hormonotherapy and has a clearly negative emotional effect that was reported by Freites-Martinez et al.[28].

In our study we also evaluated possible determinants of HRQOL and found that general quality of life, measured by FACT-G, was statistically associated with tumor stage, performance status (ECOG) and the number and severity of AEs. On the contrary, it was not associated with the type of tumor, type of treatment or duration and number of previous treatment lines received. In the multivariate analysis the variables associated with poorer quality of life in the form of a gradient were tumor stage and performance status (ECOG); female sex was also associated with poorer quality of life.

Our results highlight that despite the great advances made in tumor biology knowledge, development of multiple new drugs and improvement in supportive care, these factors remain as quality of life determinants in daily clinical practice. Cella et al. developed and validated the FACT-G questionnaire and proved that the scale was able to discriminate patients on the basis of stage of the disease and performance status [29].

They also found that FACT-G sensitivity to stage was observed not only in the total score but also in the PWB and FWB domains. Considering the fact that in our study most patients had an advanced disease (77.7% stage IV) and one of the most affected domain was the PWB domain, this data is consistent with that reported by Cella et al.

Edianto et al. assessed HRQOL in gynecologic cancer patients using FACT-G in an observational cross-sectional study. They did not find differences between treatment modalities, treatment duration and disease stage based on the total score of FACT-G [30].In their study most patients included had an early stage of the disease (57% stage I-II; 43% stage III; no patients with stage IV), in contrast to our study where most of the patients had a stage IV of the disease (77.7%), which could explain the differences regarding disease stage.

We also found that female sex was associated with a lower HRQOL. Some authors (Lee et al., Barbu et al., Andreis et al.) also reported a poorer HRQOL associated to female sex, while others did not confirm the same effect (Charles et al., Urakawa et al.). [19, 27, 31–34].

Contradictory data have also been observed in other non-oncological diseases and this could be due to different symptoms perception, associated comorbidities, degree of disease acceptance or each individual characteristics [34, 35].

Our results highlight the importance of measuring HRQOL in daily clinical practice in outpatients receiving anti-cancer treatment. In our study the lowest scores were obtained in the FWB and EWB domains; these results emphasize the fact that in our study population there could be unmet needs in those particular areas. Another finding of our study is that there are preventive measures for some of the most frequent AEs, which can clearly have an impact in HRQOL. These results have led us to reinforce joint work with oncology nursing in ambulatory care and pay more attention to provide emotional support and information about preventive measures related to AEs.

Given that we did not have data on the quality of life of these patients, the results of the present study have provided us with useful information to know in which areas it is necessary to put more emphasis and thus, together with nursing, develop recommendations for patients regarding the prevention and management of possible adverse events.

### Limitations

A limitation of the present study is that it had an observational cross-sectional design and was limited to patients from only one hospital in Pontevedra, Spain. Bias generated by using data from a single institution is also related to bias from geographical location, institutional culture, and referral patterns. Due to the cross-sectional design, the evaluation of the HRQOL was conducted only at a particular time in the patient's life, but no repeated observations or follow-up were performed. These features could have affected the results and may limit its generalizability.

## Conclusion

Our results highlight the fact that HRQOL is a subjective and multi-dimensional concept that can be influenced not only by the disease and its treatment, but also by psychological and social aspects as well as individual coping strategies. We must take into account all these factors and adapt to the specific needs of our patients. Routinely use of PRO instruments in daily clinical practice can be useful in decision making, improve patient-physician relationship, help to detect unmet needs in our patients and improve health care delivery through a comprehensive patient-centered approach.

## Data Availability

Not applicable.

## Availability of data and materials

The data that support the findings of this study are available from the corresponding author upon reasonable request.
